# Association between parental unhealthy behaviors and offspring’s cardiovascular health status: Results from a cross-sectional analysis of parent–offspring pairs in China

**DOI:** 10.3389/fped.2022.1052063

**Published:** 2023-01-06

**Authors:** Qi Ma, Jieyu Liu, Yu Wu, Mengjie Cui, Manman Chen, Tao Ma, Xinxin Wang, Di Gao, Yanhui Li, Li Chen, Ying Ma, Yi Zhang, Yanhui Dong, Yi Xing, Jun Ma

**Affiliations:** ^1^ Institute of Child and Adolescent Health, School of Public Health, Peking University; National Health Commission Key Laboratory of Reproductive Health, Beijing, China; ^2^ School of Population Medicine and Public Health, Chinese Academy of Medical Sciences/Peking Union Medical College, Beijing, China; ^3^ School of Public Health and Management, Ningxia Medical University and Key Laboratory of Environmental Factors and Chronic Disease Control, Yinchuan, China

**Keywords:** lifestyle, cardiovascular health, parents, offspring, cross-sectional study

## Abstract

**Background:**

Cardiovascular health (CVH) in children and adolescents, which might be largely influenced by parental behaviors, may affect the incidence of cardiovascular diseases in adulthood. However, few studies have been conducted to explore the associations between parental behaviors and CVH status of offspring in China.

**Methods:**

Data were obtained from a cross-sectional survey conducted in Chinese children and adolescents aged 7–18 years old, with a total of 10,043 parent–offspring pairs included. Parental behaviors included moderate to vigorous physical activity (MVPA), dietary behaviors, and weight status. The CVH status of offspring was consulted by The American Heart Association, including seven factors. The associations between parental behaviors and CVH status of offspring were evaluated by multilevel logistic regression. Stratified analyses were conducted to explore the potential modifying influence of sociodemographic factors.

**Results:**

Most of the offspring had five ideal CVH factors; only 21.04% had six to seven ideal CVH factors. Parental unhealthy behaviors were associated with high odds of nonideal CVH status of offspring. Parental overweight/obesity, insufficient MVPA, and unhealthy dietary behaviors could increase the odds of owning one to three ideal CVH factors in offspring, with corresponding odds ratios (ORs) (95% confidence interval) of 1.61 (1.32–1.96), 1.31 (1.10–1.56), and 2.05 (1.43–2.94), respectively. There was a dose–response relationship between parental single unhealthy behavior and the odds of nonideal CVH status in offspring (*P*-trend < 0.001). Offspring with overweight parents had ORs of 1.25 for nonideal CVH status, compared to offspring with normal-weight parents. Among offspring who had the same number of ideal CVH factors, the cumulative association between unhealthy behaviors of parents and offspring’s nonideal CVH status increased if parents had more unhealthy behaviors (*P*-trend < 0.001).

**Conclusions:**

Parental overweight/obesity, insufficient MVPA, and unhealthy dietary behaviors were strongly associated with CVH status in offspring. With a cumulative association, more unhealthy parental behaviors were associated with higher odds of offspring’s nonideal CVH status, suggesting that targeting parental behaviors might facilitate attainment of improving CVH status of children and adolescents.

## Introduction

Cardiovascular diseases (CVDs) are the leading cause of death around the world (1). In China, more than two in five deaths were attributed to CVDs, accounting for 45.50% and 43.16% of all deaths in rural and urban areas in 2016, which were higher than the death rate due to cancer or other diseases (2). Making efforts to improve cardiovascular health (CVH), the American Heart Association (AHA) introduced a concept of ideal CVH status, including the simultaneous presence of nonsmoking, as well as the ideal level of body mass index (BMI), physical activity, dietary behaviors, total cholesterol (TC), blood pressure (BP), and fasting plasma glucose (FPG) (3).

The ideal CVH construct appears to be a valid measure for predicting the long-term risk of major adverse cardiovascular events (4, 5). Childhood CVH status was a strong predictor for the incidence of adulthood CVDs (6). A cohort study conducted on young Finns showed that increased ideal CVH factors in children were related to lower risks of hypertension, metabolic syndrome, and carotid artery intima-media thickness (7). In order to achieve primary prevention of adulthood CVDs, substantial work must be done to improve the ideal CVH status in children and adolescents.

However, the prevalence of ideal CVH status in children and adolescents had a similar devastating trend in different countries (8, 9). Although most children and adolescents were born with ideal CVH status, their unhealthy dietary behaviors, physical inactivity, and tobacco smoking they developed gradually would largely influence their CVH status (10). It is well known that parental behaviors are crucial to developing children and adolescents’ behaviors. Previous studies had shown that parents’ diet quality and energy intake, physical activities, and the duration of screen activities were associated with those behaviors of their offspring (11, 12). In addition, the weight status between two generations was correlated according to gene and environmental factors (13, 14). Parental behaviors, therefore, may be greatly associated with the CVH status in children and adolescents.

In the Framingham Heart Study, parental CVH including BMI and nonsmoking status was positively associated with offspring’s CVH status (15). Worse maternal BMI at gestation was significantly associated with worse offspring’s CVH status in early adolescence (16). However, since multiple parental factors are reciprocally interacting, few studies explored the associations between the combined behaviors of parents including physical activity, dietary behaviors, and weight status, with the CVH status in Chinese children and adolescents. We hypothesize that parental unhealthy behaviors might increase the likelihoods of offspring’s nonideal CVH status, and the odds may continue to increase when parents with more unhealthy behaviors increase. To fill these knowledge gaps and improve the grim situation about CVDs in China, based on large and nationally representative data, we aim to explore the associations between parental behaviors and offspring’s CVH status.

## Materials and methods

### Study design and participants chosen

**Figure 1:**
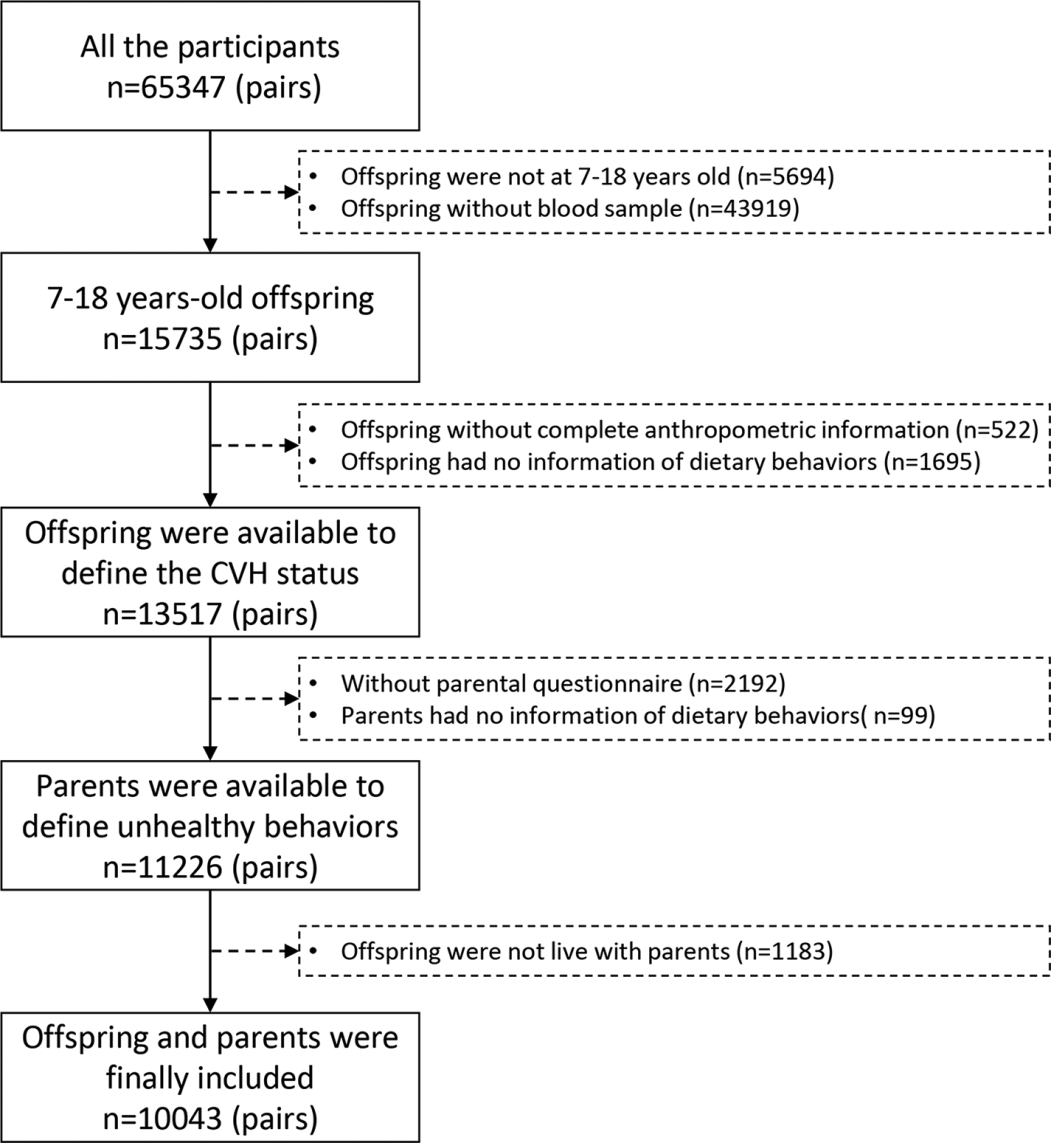
Selection process of the participants. CVH, cardiovascular health

This is a reanalysis from a previous database, which was collected from a national cross-sectional study from seven Chinese provinces in 2013. More detailed information about this study was presented previously (17). Briefly, we used multistage cluster random sampling to choose participants in seven provinces including Liaoning, Tianjin, Ningxia, Shanghai, Chongqing, Hunan, and Guangdong, which include four differently economic and geographic regions of the mainland of China. A developed and an underdevelopment city were selected in each province at the beginning. Then, 12–16 schools were randomly selected in these cities. Two classes in each grade from each school were randomly chosen subsequently, and their parents were also invited to engage in this survey. A total of 65,347 children and adolescents were included in the study. We selected 15,735 children and adolescents aged 7–18 years old randomly for blood sample examination; those who did not live with their parents or those with missing data on anthropometric measurement, parental questionnaires, and dietary behaviors were excluded. A total of 10,043 parent–offspring pairs were remained in the final analysis (Figure 1).

All survey sites used the same protocol during the implementation process, and all processes of randomization were performed by a staff who was not involved in the survey. This study was approved by the Medical Ethical Committee of Peking University (IRB No. 00001052-12072). All included students and their parents signed the written informed consents.

### Data collection in questionnaire

The childhood questionnaire was used to collect information about sex, age, residence area, inhabiting information, smoking, moderate to vigorous physical activity (MVPA), and dietary behaviors. The parental questionnaire was distributed to collect information including the primary respondent of this questionnaire, offspring’s birth weight, single-child status, breastfeeding status, parental self-reported height and weight, highest educational attainment, MVPA, dietary behaviors, and family history of chronic diseases. For birth weight classification, low birth weight and high birth weight were defined as weight <2,500 and ≥4,000 g at delivery, respectively (18). All other information collected in the questionnaire is presented in Supplementary Tables S1, S2.

The frequency (days) and amount (serving per day) were investigated for dietary behaviors, including the total consumption of vegetables, fruits, sugar-sweetened beverages (SSBs), and meat over the past 7 days. One serving of vegetables/fruits was defined as the size of an ordinary adult’s closed fist with roughly 200 g (19); one serving of SSBs was determined as a canned beverage (approximately 250 ml), including orange juice, Nutrition Express, Red Bull, Coca-Cola, and Sprite (20); one portion of meat equaled the size of an adult’s palm (approximately 100 g) (21). We calculated the daily dietary intake as (days of consumption × servings in those days)/7. In addition, the frequency (times) over the past 7 days was investigated for fried foods. As set in questionnaires, fried food consumption referred to fried chicken, deep-fried dough sticks, and other common fried foods.

The self-administered child questionnaires were delivered to students (except grades 1–3) in a class meeting by the trained school teacher. Child questionnaires of children grades 1–3 and parental questionnaires were filled by students’ fathers or mothers. Trained project members provided appropriate guidance and interpreted all the questions as effectively as possible. Three percent of the questionnaires were rechecked within 1 week for the same participants.

### Anthropometric measurements

Anthropometric measurements were conducted to measure the height, weight, and BP of children by trained investigators in schools, according to the principles described in the Chinese Students’ Physical Fitness and Health Survey Report 2010 (22). Children were asked to stand straight in light clothing and without shoes. Height was measured using the portable stadiometer with 0.1 cm precision, and weight was measured to the nearest 0.1 kg by a Lever-type might scale. BP was measured in the right arm by a Riva-Rocci sphygmomanometer. Every indicator was measured twice, and the average of the two measurements was used for final analyses. BMI was calculated as the weight (kg) divided by the square of the height (m^2^).

### Blood sample collection and detection

After overnight fasting for 8–12 h, 5 ml venous blood samples were collected for each child or adolescent into Ethylene Diamine Tetraacetic Acid (EDTA) vacuum tubes. Children were asked to rest for at least 10 min before blood sample collection. Blood specimens were transported in a chilled insulated container immediately, centrifuged at 3,000 rpm for 10 min, and then frozen at −80 °C before transported in dry ice to the laboratory in Beijing, where the samples were stored at −80 °C before laboratory detection. Blood tests were performed by an autoanalyzer (Hitachi 7080, Japan). FPG and TC were tested using the glucose oxidase method, enzymatic method, and clearance method. All biochemical analyses on blood were carried out at a biomedical analyses company, which is accredited by Peking University (17).

### Definition of ideal CVH of offspring

CVH status of offspring included smoking status, BMI, MVPA, dietary behaviors, BP, FPG, and TC. According to Chinese standards for overweight/obesity (23) and BP (24), The Chinese Dietary Guideline (2016) (25), and AHA (3), we defined the ideal CVH factors as follows (Supplementary Table S3). Children and adolescents meeting 6–7 ideal CVH factors were defined as having ideal CVH status (26), and others were defined as having nonideal CVH status. Participants of nonideal CVH status were divided into three groups according to the numbers of ideal CVH factors they had: 1–3 ideal CVH factors, 4 ideal CVH factors, and 5 ideal CVH factors.

### Definition of unhealthy behaviors of parents

Unhealthy behaviors for parents included unhealthy dietary behaviors, insufficient MVPA, and overweight/obesity defined by BMI. All the information about behaviors represented one side of the parents who filled the questionnaire. Parental unhealthy dietary behaviors was determined as <4 healthy dietary factors (the factors was the same to children); insufficient MVPA referred to <150 min/week according to Physical Activity Guidelines for the Chinese Population (2021) (27) and AHA (3); overweight was defined as BMI ≥ 24 and BMI < 28 kg/m^2^, and obesity was defined as BMI ≥ 28 kg/m^2^ according to the criteria established by the Working Group on Obesity in China (28). The combined unhealthy behaviors of parents were defined by summing the number of single unhealthy behavior.

### Statistical analysis

Descriptive analyses were used to present the basic information of the offspring and parents. Differences by sex were examined using Student’s t-test for continuous variables and Pearson’s *χ*^2^ test for categorical variables.

We used a multilevel logistic regression model to eliminate the effects of province-cluster confounders, with provinces set as a random factor in the model. All the outcomes were treated as disordered, not satisfying the test of parallel lines. To explore the association between different unhealthy behaviors of parents and nonideal CVH status of offspring, parental single behavior was examined as two levels (healthy and unhealthy) and the CVH status of offspring was examined as a multiple-category outcome (had 1–3, 4, 5, and 6–7 ideal CVH factors), using pairwise regression. To assess the dose–response association between single unhealthy behavior of parents and offspring’s nonideal CVH status, we examined the single unhealthy behavior of parents as multiple-category outcomes and CVH status of offspring as two variable categories (ideal CVH status and nonideal status, respectively). We also derived a score of parental combined unhealthy behaviors by summing the number of single unhealthy behavior and evaluated the cumulative association between parental combined unhealthy behaviors and offspring’s nonideal CVH status. To assess the influence of sociodemographic factors on the associations between parental unhealthy behaviors and offspring’s CVH status, stratified analyses were additionally conducted. *P* for trend values were calculated by including the median of each category of single/combined unhealthy behavior of parents as a continuous variable in the model; the interactions between parental unhealthy behaviors and sociodemographic factors were examined using the likelihood ratio test, with a comparison of the log likelihood of the two models with and without the interaction terms. All the models were adjusted for confounders including provinces, family history of chronic diseases, family monthly income, offspring’s age, sex, residence area, birth weight, single-child status, breastfeeding, and parental highest educational attainment. Odds ratio (ORs) and 95% confidence interval (95% CI) were calculated and represented.

The number of each missing variable was approximately 30–884, less than 9% of the total participants (Supplementary Table S4). We used linear interpolation and median to refill the missing data of continuous and categorical variables *via* the section of Replace Missing Values in SPSS 25.0. To overcome potential limitations, two separate sensitivity analyses were also conducted: (1) all the incomplete data were excluded to evaluate the bias that the refilling method may have; (2) participants with parental BMI < 18.5 kg/m^2^ [which was defined as thinness (28)] was excluded.

All analyses were performed using IBM SPSS Statistics software (version 25.0, SPSS, IBM, Armonk, NY, United States). A two-sided *P*-value <0.05 was considered statistically significant.

## Result

### Baseline characteristics

Table 1 shows the basic characteristics of 10,043 parent–offspring pairs included in the present study. Offspring were 11.22 years old on average, and 44.69% of them were boys. Most of them had five ideal CVH factors, and only 21.04% had six to seven ideal CVH factors. Among all the CVH factors, healthy dietary behaviors accounted for the lowest proportion (9.06%), while nonsmoking status accounted for the highest proportion (98.91%), which was more evident in girls (*P *< 0.001). Of their parents, more than 50% had the educational attainment of senior high school or above, 47.60% of them had two unhealthy behaviors and only 3.46% had completely combined healthy behaviors.

**Table 1:** Descriptive characteristics of the study population stratified by sex.

Variable	Total	Boys	Girls	*P*-value
Provinces, No. (%)				
Hunan	757 (7.54)	397 (8.04)	360 (7.05)	0.086
Ningxia	827 (8.23)	380 (7.70)	447 (8.76)	
Tianjin	2,050 (20.41)	1,005 (20.35)	1,045 (20.47)	
Chongqing	1,215 (12.10)	592 (11.99)	623 (12.20)	
Liaoning	1,600 (15.93)	777 (15.74)	823 (16.12)	
Shanghai	1,769 (17.61)	908 (18.39)	861 (16.87)	
Guangzhou	1,825 (18.17)	879 (17.80)	946 (18.53)	
Residence area, No. (%)				
Urban	5,564 (55.40)	2,273 (55.14)	2,841 (55.65)	0.609
Rural	4,479 (44.60)	2,215 (44.86)	2,264 (44.35)	
Family history of chronic diseases, No. (%)				
No	3,594 (35.79)	1,828 (37.02)	1,766 (34.59)	0.011
Yes	6,449 (64.21)	3,110 (62.98)	3,339 (65.41)	
Family monthly income (RMB), No. (%)				
<12,000	6,085 (60.59)	2,956 (59.86)	3,129 (61.29)	0.199
≥12,000	900 (8.96)	437 (8.85)	463 (9.07)	
Do not know/missing data	3,058 (30.45)	1,545 (31.29)	1,513 (29.64)	
Offspring characteristics				
Age, mean ± SD	11.22 ± 3.11	11.12 ± 3.08	11.32 ± 3.14	0.002
Breastfeeding, No. (%)				
Yes	8,646 (86.09)	4,229 (85.64)	4,417 (86.52)	0.202
No	1,397 (13.91)	709 (14.36)	688 (13.48)	
Birth weight (g), No. (%)				
<2,500	169 (1.68)	78 (1.58)	91 (1.78)	<0.001
2,500–4,000	8,083 (80.48)	3,851 (77.99)	4,232 (82.90)	
≥4,000	1,791 (17.83)	1,009 (20.43)	782 (15.32)	
Single children, No. (%)				
Yes	6,851 (68.22)	3,553 (71.95)	3,298 (64.60)	<0.001
No	3,192 (31.78)	1,385 (28.05)	1,807 (35.40)	
BMI, mean ± SD	18.84 ± 3.93	19.16 ± 4.15	18.54 ± 3.68	<0.001
Number of healthy dietary factors, No. (%)				
0–3	9,133 (90.94)	4,510 (91.33)	4,623 (90.56)	0.177
4–5	910 (9.06)	428 (8.67)	482 (9.44)	
Smoking status, No. (%)				
Smoking in past 30 days	109 (1.09)	73 (1.48)	36 (0.71)	<0.001
Nonsmoking in past 30 days	9,934 (98.91)	4,865 (98.52)	5,069 (99.29)	
MVPA (min/day), No. (%)				
<60	6,880 (68.51)	3,141 (63.61)	3,739 (73.24)	<0.001
≥60	3,163 (31.49)	1,797 (36.39)	1,366 (26.76)	
TC (mmol/L), No. (%)				
≥5.17	557 (5.55)	249 (5.04)	308 (6.03)	0.030
<5.17	9,486 (94.45)	4,689 (94.96)	4,797 (93.97)	
FPG (mmol/L), No. (%)				
≥5.60	213 (2.12)	152 (3.08)	61 (1.19)	<0.001
<5.60	9,830 (97.88)	4,786 (96.92)	5,044 (98.81)	
BP, No. (%)				
High	2,466 (24.55)	1,378 (27.91)	1,088 (21.31)	<0.001
Normal	7,577 (75.45)	3,560 (72.09)	4,017 (78.69)	
Weight status, No. (%)				
Overweight/obesity	2,555 (25.44)	1,537 (31.13)	1,018 (19.94)	<0.001
Normal	7,488 (74.56)	3,401 (68.87)	4,087 (80.06)	
Number of ideal CVH factors, No. (%)				
1–3	775 (7.72)	471 (9.54)	304 (5.95)	<0.001
4	2,525 (25.14)	1,321 (26.75)	1,204 (23.58)	
5	4,630 (46.10)	2,102 (42.57)	2,528 (49.52)	
6–7	2,113 (21.04)	1,044 (21.14)	1,069 (20.94)	
Parental characteristics				
Parental highest educational attainment, No. (%)				
Junior high school or below	4,666 (46.46)	2,311 (46.80)	2,355 (46.13)	0.502
Senior high school or above	5,377 (53.54)	2,627 (53.20)	2,750 (53.87)	
Unhealthy behaviors, No. (%)				
0	347 (3.46)	167 (3.38)	180 (3.53)	0.466
1	3,881 (38.64)	1,947 (39.43)	1,934 (37.88)	
2	4,780 (47.60)	2,321 (47.00)	2,321 (47.00)	
3	1,035 (10.31)	503 (10.19)	503 (10.19)	

SD, standard deviation; BMI, body mass index; MVPA, moderate to vigorous physical activity; TC, total cholesterol; FPG, fasting plasma glucose; BP, blood pressure; CVH, cardiovascular health.

P-value was calculated by T-test for continuous variables and by Pearson's chi-squared test for categorical variables.

### Epidemiology of single behavior of parents and CVH status of offspring

**Figure 2:**
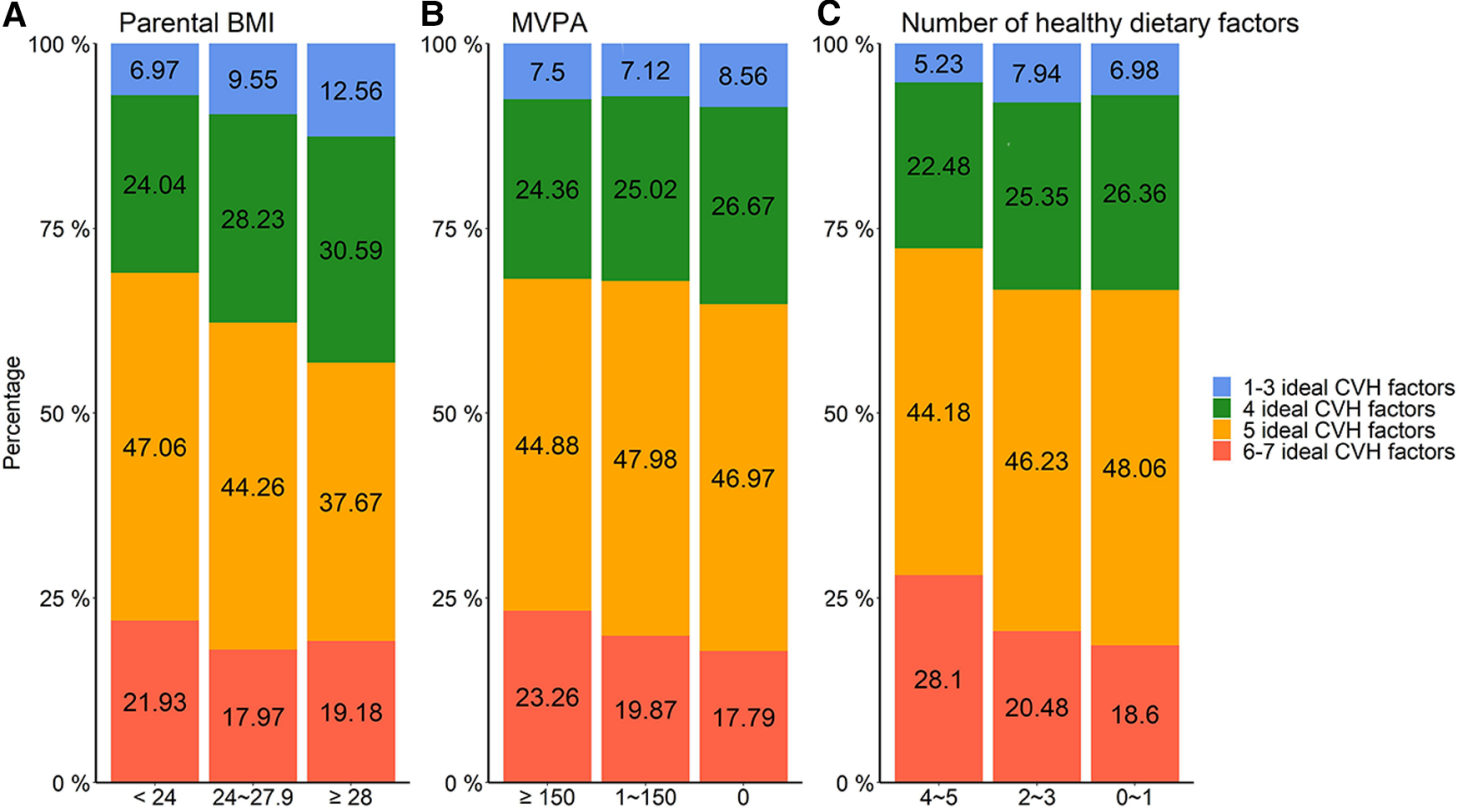
Distribution of participants in different CVH status according to parental behaviors: (**A**) parental BMI, (**B**) MVPA, and (**C**) number of healthy dietary factors. CVH, cardiovascular health; BMI, body mass index; MVPA, moderate to vigorous physical activity.

With the increase of parents’ BMI, the decrease of MVPA time, and the number of healthy dietary factors, the percentages of offspring with one to three and four ideal CVH factors increased, and the percentages of six to seven ideal CVH factors decreased (Figure 2 and Supplementary Table S5).

### Association between single unhealthy behavior of parents and offspring’s nonideal CVH status

Table 2 presents the associations between single unhealthy behavior of parents and offspring’s nonideal CVH status. Parental overweight/obesity, insufficient MVPA, and unhealthy dietary behaviors were associated with higher odds of offspring’s nonideal CVH status. Parental overweight/obesity, insufficient MVPA, and unhealthy dietary behaviors could increase the odds of owning one to three ideal CVH factors in offspring, with corresponding ORs (95% CI) of 1.61 (1.32–1.96), 1.31 (1.10–1.56), and 2.05 (1.43–2.94).

**Table 2:** Association between single unhealthy behavior of parents and offspring’s different groups of nonideal CVH status.

Parental unhealthy behaviors	1–3 ideal CVH factors, OR (95% CI)	4 ideal CVH factors, OR (95% CI)	5 ideal CVH factors, OR (95% CI)	6–7 ideal CVH factors, OR (95% CI)
Parental overweight/obesity (vs. normal weight)	**1.61(1.32–1.96)**	**1.41 (1.22–1.62)**	1.11 (0.97–1.26)	1 (Reference)
Insufficient MVPA (vs. sufficient)	**1.31 (1.10–1.56)**	**1.33 (1.18–1.50)**	**1.29 (1.16–1.44)**	1 (Reference)
Unhealthy dietary behaviors (vs. healthy)	**2.05 (1.43–2.94)**	**1.57 (1.27–1.95)**	**1.42 (1.18–1.70)**	1 (Reference)

A multilevel logistic regression model was used, with pairwise regression, respectively. Data adjusted for provinces, family history of chronic diseases, family monthly income, offspring's sex, age, resident area, birth weight, single-child status, breast feeding status, and parental highest education attainment.

CVH, cardiovascular health; MVPA, moderate to vigorous physical activity; OR, odds ratio; CI, confidence interval.

The bold values indicated the ORs were statistically significant.

**Figure 3:**
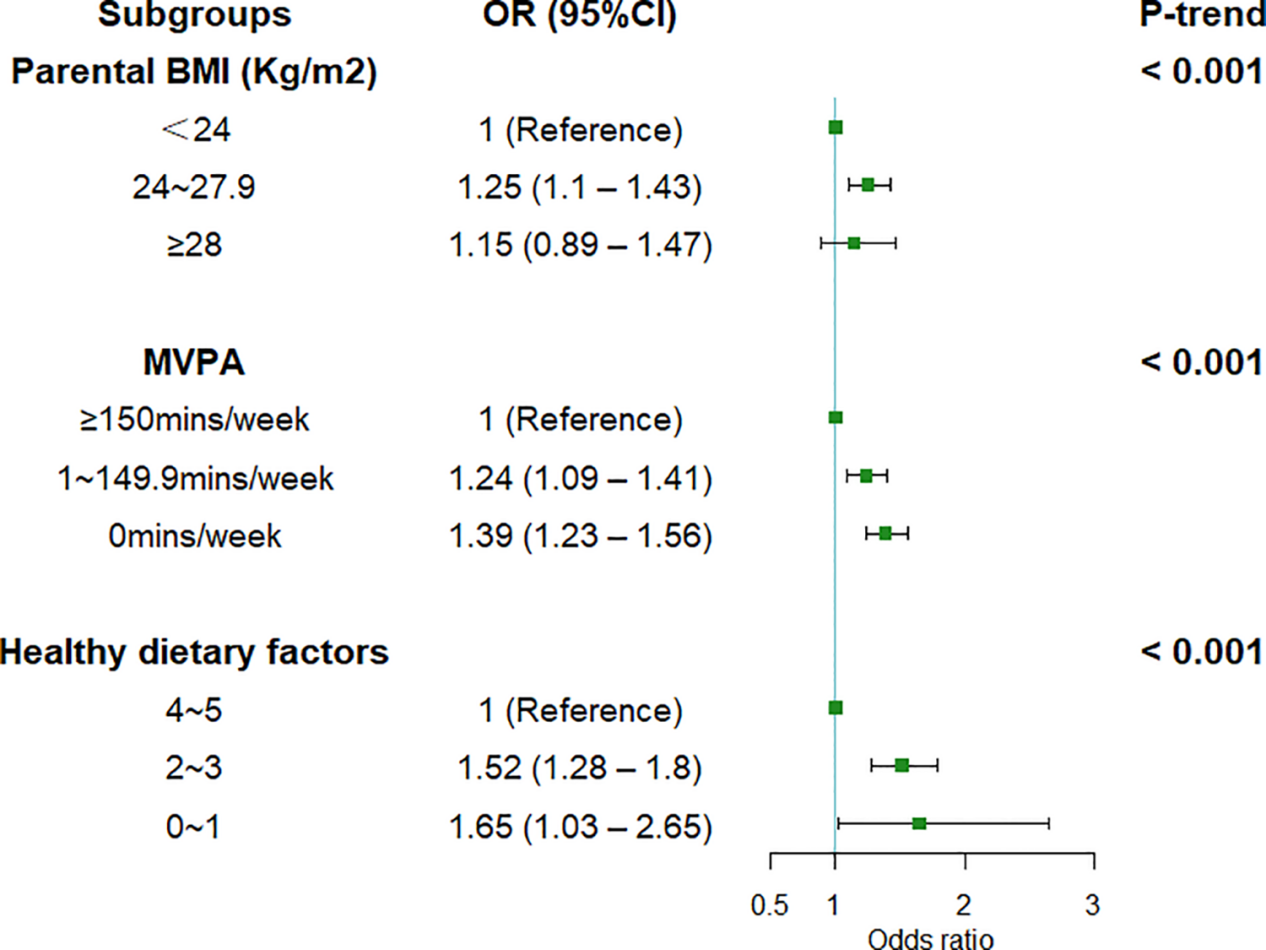
Association between different levels of single behavior of parents and offspring’s nonideal CVH status. Error bar was 95% CI. Multilevel logistic regression was used, and CVH status was defined as nonideal status or ideal CVH status. Data adjusted for provinces, resident area, family history of chronic diseases, family monthly income, offspring’s sex, age, birth weight, single-child status, breast feeding status, and parental highest education attainment. CVH, cardiovascular health; BMI, body mass index; MVPA, moderate to vigorous physical activity; OR, odds ratio; CI, confidence interval.

Figure 3 presents a dose–response relationship between single unhealthy behavior of parents and the odds of nonideal CVH status of offspring (all the *P*-trend < 0.001). Offspring with overweight parents had 25% higher odds (OR = 1.25, 95% CI = 1.10–1.43) for nonideal CVH status compared to offspring whose parents had a normal weight. Parental unhealthy dietary behaviors could increase the odds of offspring’s nonideal CVH status by 52% (OR = 1.52, 95%CI = 1.28–1.8) and 65% (OR = 1.65, 95%CI = 1.03–2.65).

### Association between combined unhealthy behaviors of parents and offspring’s nonideal CVH status

**Figure 4:**
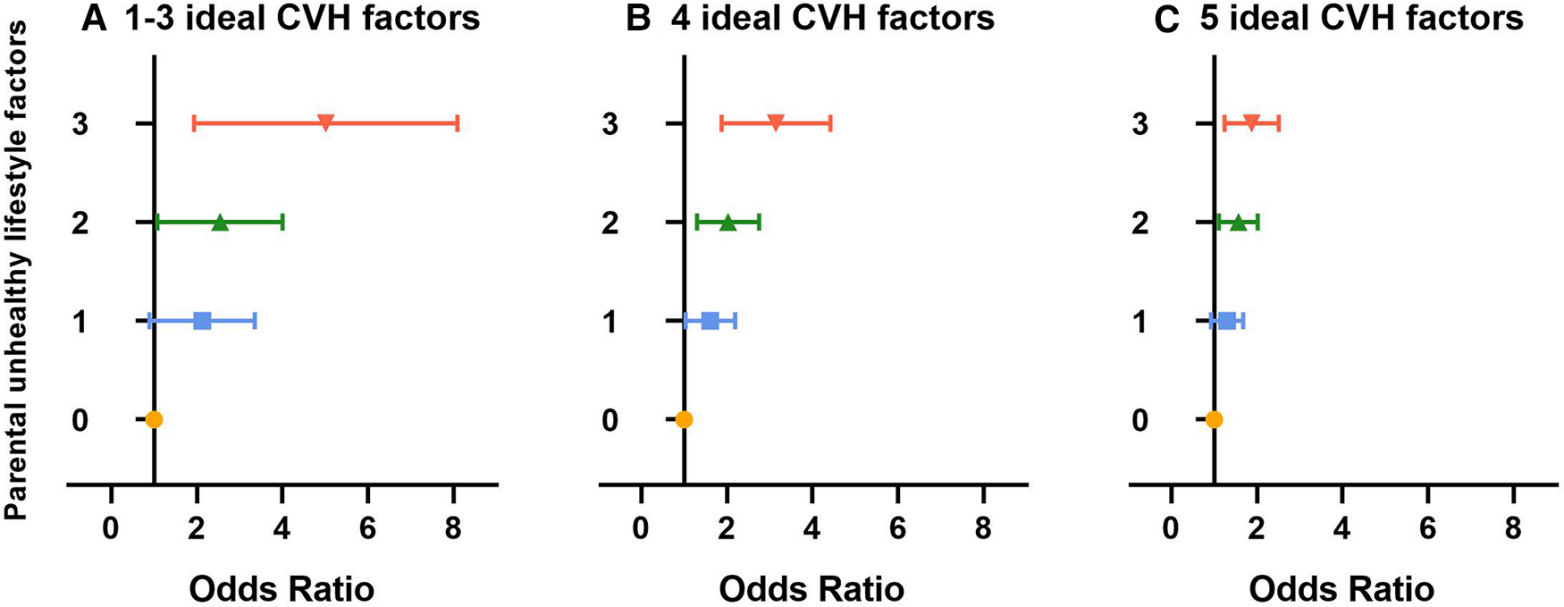
Association between combined unhealthy behaviors of parents and offspring's different groups of nonideal CVH status: (**A**) 1-3 ideal CVH factors, (**B**) 4 ideal CVH factors, (**C**) 5 ideal CVH factors. Error bar was 95% CI. The combined behaviors of parents were examined as riable (vs. ideal CVH status). Data adjusted for provinces, resident area, family history of chronic diseases, family monthly incng's sex, age, birth weight, single-child status, breast feeding status, and parental highest education attainment. CVH, cardiovascular health.

The ORs in different groups of offspring’s nonideal CVH status stratified by the number of parental unhealthy behaviors are presented in Figure 4 and Supplementary Table S6. The odds of nonideal CVH status in offspring with parents who had combined unhealthy behaviors were higher than those whose parents had completely healthy behaviors. Among offspring in the same group of nonideal CVH status, the odds of nonideal CVH status in offspring would increase if the number of parental unhealthy behaviors increased (all the *P*-trend < 0.001). For example, compared to offspring with parents who had completely healthy behaviors, offspring with parents who had three, two, and one unhealthy behavior had ORs (95% CI) of 3.13 (2.16–4.53), 2.20 (1.59–3.05), and 1.65 (1.19–2.29), respectively, for owning four ideal CVH factors.

Notably, the associations between parental combined unhealthy behaviors and offspring’s nonideal CVH status did not change stratified by sociodemographic factors (Supplementary Table S7).

### Sensitivity analysis

Associations between parental unhealthy behaviors and offspring’s nonideal CVH status were robust in all sensitivity analyses, including the following: (1) all the incomplete data were excluded (Supplementary Tables S8–S10); (2) participants with parental BMI < 18.5 kg/m^2^ were excluded (Supplementary Tables S11–S13).

## Discussion

To our knowledge, parental unhealthy behaviors play a crucial role in the offspring’s CVH status in China. Parental overweight/obesity, insufficient MVPA, and unhealthy dietary behaviors were independently strongly associated with nonideal CVH status of offspring, with dose–response associations presented in all behaviors. We also found that the odds of nonideal CVH status of offspring continued to increase as the number of parental unhealthy behaviors increased. Our study emphasized the importance of family-focused community-based prevention programs, proposing that parental behaviors should receive more attention in childhood obesity prevention policies.

In the present study, considering parental behaviors as part of factors of CVH, both single and combined unhealthy behaviors of parents were associated with nonideal CVH status in offspring. One previous study indicated that worse maternal CVH status (included five factors excepted dietary behaviors and MVPA) at gestation was significantly associated with offspring’s worse CVH status in early adolescence (16), and a strong correlation between CVH factors of parents and their offspring was also found when they were at similar ages (15). In the present study, we extended the contents of offspring’s CVH status, and only three kinds of behaviors (overweight/obesity, MVPA, and dietary behaviors) for parental CVH status were selected. Thus, the present study paid more attention on the associations between modifiable behaviors of parents and offspring’s CVH status. Muchira et al. analyzed the association between parental CVH factors and time to onset of CVDs in the offspring and found that offspring would have a longer CVD-free survival if their parents had ideal CVH status (26). Considering one’s ideal CVH status could predict his risk of major CVDs in the future and affect the CVH status of his offspring (4, 5), our study may provide new evidence from parental CVH status to offspring’s future CVDs. However, we did not detect significant differences in associations between parental combined unhealthy behaviors and offspring’s CVH status when stratified by sociodemographic characteristics, and more research studies are urgently needed to verify our conclusions.

The close relationships of multiple lifestyle behaviors between two generations are the potential explanation of the findings. It was well acknowledged that the diet quality and energy intake of parents and their offspring were closely associated in the family (11), same as the pattern of physical activities (12). In addition, parental BMI was a predictor for offspring BMI, exactly as the genetic and environmental factors explicated (13, 29, 30). Family habits contribute the most in modeling children’s lifestyle behaviors as they represent an important moment of parent–child interaction (31), which could also explain the dose–response/cumulative association between single/combined unhealthy behaviors of parents and nonideal CVH status of offspring. In detail, mothers, who were preoccupied with their own weight and eating habit, reported higher levels of encouraging their daughters to lose weight thus leading to daughters’ restrained eating habits (32). Parental behaviors may have a broad and long-term influence on offspring’s CVH status (33); the underlying potential mechanism might be attributed to the following reasons. Offspring loaded heavily on fried foods, SSBs, and refined grain had increased risks of impaired glucose tolerance, dyslipidemia, and hypertension (34, 35); the total blood volume increases with increasing BMI through central (renin-angiotensin and sympathetic systems) and peripheral (e.g., baroreceptors and autonomic dysregulation) mechanisms (36); and insufficient MVPA might disrupt the lipid metabolism through inactivating the lipoprotein lipase and preventing C-reactive protein reduction (37). Thus, although dietary behaviors, MVPA, and weight status of offspring could be influenced by parental behaviors directly, changes in these behaviors with the passage of time could disturb the body homeostasis and result in abnormal BP, TC, and FPG (38–40). In addition, overweight and obesity have long been recognized as key indicators for cardiovascular diseases (41). The potential abnormal metabolic and cardiovascular indicators could pass down to offspring through epigenetic pathways if parents have been at overweight/obesity status for a long period (42, 43). Although phenotype was based on the heritability, there are differences between family-cluster phenotype and heritability owing to the interaction of gene and environment (44). For instance, frequent physical activity could mitigate the impact of genetic factors on the development of high BMI and waist circumference (45); and the specific contents of CVH status between two generations were different owing to the environment (15). Family-targeted improvement strategies should, therefore, be included to promote their offspring’s CVH status, and comprehensive interventions should be considered and encouraged at family’s dimensions.

Our study has practical values for public health. Since parental behaviors are modifiable and they are major contributors to ideal CVH status, only 15% of ideal CVH status is heritable (15). Parental healthy behaviors play a prominent part in the improving the CVH status of offspring under the context of Chinese culture of valuing family connections and supervision. We emphasize that even mild improvements of parental behaviors will benefit their offspring’s CVH status in the context of the dose–response relationship. In addition, as previously described (16), both single and combined unhealthy behaviors of parents would influence offspring’s nonideal CVH status, public service should encourage all parents to improve their healthy behaviors rather than only focus on those parents whose offspring had worse CVH status, and the whole family should be encouraged to be involved in the educational interventions to prevent unhealthy behaviors in children and adolescents.

The strength of the present study was the large sample size collected from seven Chinese provinces including all the economic and geographic regions, which made our findings be generalized to all Chinese school-age population. In addition, we used high-quality CVH factor measurements for offspring, including standardized protocols and repeated operations for anthropometric and BP measurements, as well as professional blood sample detection. In addition, we conducted multiple analyses for both parental unhealthy behaviors and offspring’s CVH status, making the results become more reliable. However, several limitations should be mentioned. First, information during pregnancy was absent, including maternal disease and prenatal care, which limited us to make further inferences. Second, the self-reported physical activity, parental height, and weight might not be as accurate as measured directly. However, self-reported physical activity has been proved to be useful in large epidemiological studies (46), and self-reported height and weight in adults were demonstrated to be highly reliable (47, 48). Third, potential selection bias might influence the results, but we adjusted for socioeconomic and sociodemographic variables in order to avoid the possible deviations to a large extent. In addition, we collected only one side of the parents’ behaviors, and thus whether maternal or paternal behaviors were more important on offspring CVH status could not be speculated. Finally, we only examined the associations between some of the parental behaviors (overweight/obesity, MVPA, and dietary behaviors) and offspring’s CVH status but did not include parental smoking status due to the limited information of the database. Prospective research studies examining more comprehensive behaviors’ regulations on such parent–offspring associations are still needed.

## Conclusion

Parental overweight/obesity, insufficient MVPA, and unhealthy dietary behaviors were strongly associated with offspring’s nonideal CVH status, with dose–response associations. In addition, more unhealthy parental behaviors are associated with higher risks of childhood nonideal CVH status. Targets to improve parental lifestyle behaviors might facilitate the attainment of improving CVH status of offspring, and the whole family should be encouraged to be involved in the educational interventions to benefit to their offspring’s lifelong cardiovascular health.

## Data Availability

The raw data supporting the conclusions of this article will be made available by the authors, without undue reservation.
