# Supplementary material for: Association between parental unhealthy behaviors and offspring’s cardiovascular health status: Results from a cross-sectional analysis of parent–offspring pairs in China
Source: Front Pediatr. 2023 Jan 6;10:1052063. doi: 10.3389/fped.2022.1052063 (PMC9853557; doi:10.3389/fped.2022.1052063)
Supplement: Supplementary file 1 [file Datasheet1.docx]

**Supplementary Materials**

**Table S1**. Information on covariates in the offspring’s questionnaires

| Covariates | Questions | Answers |
| --- | --- | --- |
| Residence area | “Where your family is located?” | 1.Rural 2.Urban |
| Inhabiting information | “Do you live with your parents in this semester?” | 1.Yes 2.No |
| Smoking status | “Have you smoked in the past 30 days?” | 1.Yes 2. No |
| MVPA | “How many days, over the past 7 days, have you had moderate/vigorous physical activity? How long in one day ?” | days minutes |
| SSBs/Meat/Fruit/Vegetable | “How many days, over the past 7 days, have you eaten meat/fruit/vegetable or drunk SSBs? How many servings in one day?” | days servings |
| Fried foods | “How many times, over the past 7 days, have you eaten fried foods?” | times |

Note: MVPA, moderate to vigorous physical activity; SSBs, sugar-sweetened beverages

**Table S2**. Information on covariates in the parental questionnaires

| Covariates | Questions | Answers |
| --- | --- | --- |
| Primary respondent | -- | 1. Father 2. Mother 3. Both father and mother 4. Grandparent 5. Others |
| Birthweight | “What is your child’s birthweight?” | Kg |
| Single–child status | “Is your child a single child?” | 1.Yes 2.No |
| Breastfeeding | “Is your child breastfeeding?” | 1.Yes 2.No |
| Parental educational attainment | “What is the father’s educational attainment?”  “What is the mother’s educational attainment?” | 1.Primary school or below  2.Junior high school and senior high school  3.Junior college or above |
| Parental height/weight | “What is the father’s height/weight?”  “What is the mother’s height/weight?” | cm  Kg |
| MVPA | “How many days, over the past 7 days, have you had MVPA? How long in one day ?” | days minutes |
| SSBs/Meat/Fruit/Vegetable | “How many days, over the past 7 days, have you eaten meat/fruit/vegetable or drunk SSBs? How many servings in one day?” | days servings |
| Fried foods | “How many times, over the past 7 days, have you eaten fried foods?” | times |
| Family history of chronic diseases | “Is there anyone in your family had disease history of hypertension/diabetes/cardiopathy/ cerebrovascular disease/obesity?” | 1.Mother 2.Father 3.Grandparent 4.Brother 5.Sister 6.Aunt 7.Uncle 8.No one |

Note: MVPA, moderate to vigorous physical activity; SSBs, Sugar-sweetened beverages

**Table S3**. Definition of ideal CVH factors.

| CVH factors | Definition of ideal CVH factors |
| --- | --- |
| Smoking status | Never tried; never smoked the whole cigarette |
| BMI | ≤ the “overweight” threshold of the corresponding sex and age group |
| MVPA | ≥60 mins/day |
| Dietary Behaviors | Satisfy 4–5 factors   - Vegetables: ≥4 servings per day - Fruits: ≥3 servings per day - Meat: <3 servings per day - Sugar-sweetened beverages (SSBs): <2 servings per day - Fried foods: <2 times per week |
| TC | ≤5.18 mmol/L |
| BP | <90th percentile of the corresponding sex, age and height group |
| FPG | <5.6 mmol/L |

Notes: BMI, body mass index; MVPA, moderate to vigorous physical activity; SSBs, Sugar-sweetened beverages; TC, total cholesterol; FPG, fasting plasma glucose; BP, blood pressure; CVH, cardiovascular health.

**Table S4**. Frequency of each missing variable.

| Variables | Missing records  n (%) | Effective records  n (%) |
| --- | --- | --- |
| ***Offspring variables*** |  |  |
| Breastfeeding | 30 (0.3) | 10013 (99.7) |
| Birthweight | 569 (5.7) | 9474 (94.3) |
| MVPA | 426 (4.2) | 9617 (95.8) |
| SSBs consumption/day | 344 (3.4) | 9699 (96.6) |
| Meat consumption/day | 158 (1.6) | 9885(98.4) |
| Vegetables consumption/day | 130 (1.3) | 9913 (98.7) |
| Fruits consumption/day | 133 (1.3) | 9910 (98.7) |
| Frequency of eating fried foods | 100 (1.0) | 9943 (99.0) |
| Smoking status | 96 (1.0) | 9947 (99.0) |
| DBP | 42 (0.4) | 10001 (99.6) |
| SBP | 41 (0.4) | 10002 (99.6) |
| ***Parental variables*** |  |  |
| MVPA | 569 (5.7) | 9474 (94.4) |
| SSBs consumption/day | 324 (3.2) | 9719 (96.8) |
| Meat consumption/day | 249 (2.5) | 9794 (97.5) |
| Vegetables consumption/day | 179 (1.8) | 9864 (98.2) |
| Fruits consumption/day | 177 (1.8) | 9866 (98.2) |
| Frequency of eating fried foods | 70 (0.7) | 9973 (99.3) |
| Height | 192 (1.9) | 9851 (98.1) |
| Weight | 295 (2.9) | 9748 (97.1) |
| Highest educational attainment | 884 (8.8) | 9159 (91.2) |

Notes: SSBs: sugar-sweetened beverages; MVPA: moderate to vigorous physical activity; DBP: diastolic blood pressure; SBP: systolic blood pressure

**Table S5**. Distribution of participants in different CVH status stratified by parental lifestyle factors.

| Parental lifestyle factors | 1–3 ideal CVH factors | 4 ideal CVH factors | 5 ideal CVH factors | 6–7 ideal CVH factors | P-Value |
| --- | --- | --- | --- | --- | --- |
| Parental BMI (No (%)) |  |  |  |  | <0.001 |
| < 24 Kg/m^2^ | 533 (6.97) | 1838 (24.04) | 3598 (47.06) | 1677 (21.93) |  |
| 24~27.9 Kg/m^2^ | 187 (9.55) | 553 (28.23) | 867 (44.26) | 352 (17.97) |  |
| ≥28 Kg/m^2^ | 55 (12.56) | 134 (30.59) | 165 (37.67) | 84 (19.18) |  |
| Parental MVPA (No (%)) | |  |  |  | <0.001 |
| ≥150 mins/week | 388 (7.50) | 1260 (24.36) | 2321 (44.88) | 1203 (23.26) |  |
| 1~150 min/week | 148 (7.12) | 520 (25.02) | 997 (47.98) | 413 (19.87) |  |
| 0 min/week | 239 (8.56) | 745 (26.67) | 1312 (46.97) | 497 (17.79) |  |
| Number of healthy dietary factors (No (%)) | |  |  |  | <0.001 |
| 4~5 | 40 (5.23) | 172 (22.48) | 338 (44.18) | 215 (28.10) |  |
| 2~3 | 726 (7.94) | 2319 (25.35) | 4230 (46.23) | 1874 (20.48) |  |
| 0~1 | 9 (6.98) | 34 (26.36) | 62 (48.06) | 24 (18.60) |  |

Notes: Pearson's Chi-squared test was used to test the differences of participants’ distribution in different CVH status stratified by parental lifestyle factors. CVH, cardiovascular health; BMI, body mass index; MVPA, moderate to vigorous physical activity.

**Table S6**. Trend analysis of association between combined unhealthy behaviors of parents and offspring’s different groups of non-ideal CVH status.

| Number of parental unhealthy lifestyle factors | 1–3 ideal CVH factors  OR（95%CI） | 4 ideal CVH factors  OR（95%CI） | 5 ideal CVH factors  OR（95%CI） | 6–7 ideal CVH factors  OR（95%CI） |
| --- | --- | --- | --- | --- |
| 0 | 1 (Reference) | 1 (Reference) | 1 (Reference) | 1 (Reference) |
| 1 | **1.82 (1.05 – 3.13)** | **1.65 (1.19 – 2.29)** | **1.31 (1.01 – 1.69)** | 1 (Reference) |
| 2 | **2.40 (1.40 – 4.14)** | **2.20 (1.59 – 3.05)** | **1.68 (1.30 – 2.18)** | 1 (Reference) |
| 3 | **4.33 (2.42 – 7.75)** | **3.13 (2.16 – 4.53)** | **1.90 (1.40 – 2.58)** | 1 (Reference) |
| P–Trend | <0.001 | <0.001 | <0.001 | –– |

Notes: The bold values indicated the ORs were statistically significant. A multilevel logistic regression model was used, with pairwise-regression, respectively. P for trend values were calculated by including the combined unhealthy behavior of parents as a continuous variable in the model. Adjusted for provinces, family history of chronic diseases, family monthly income, offspring's sex, age, resident area, birthweight, single-child status, breast feeding status, and parental highest education attainment. CVH, cardiovascular health.

**Table S7**. Association between combined unhealthy behaviors of parents and offspring’s non-ideal CVH status stratified by social and demographic factors.

| Variable | Group 1  OR（95%CI） | Group 2  OR（95%CI） | Group 3  OR（95%CI） | P-trend | P-interaction |
| --- | --- | --- | --- | --- | --- |
| *Sex* |  |  |  |  | 0.914 |
| Boys | **2.43 (1.83 – 3.23)** | **1.90 (1.49 – 2.42)** | **1.44 (1.13 – 1.84)** | <0.001 |  |
| Girls | **2.39 (1.60 – 3.57)** | **1.80 (1.29 – 2.52)** | 1.40 (1.00 – 1.96) | <0.001 |  |
| *Age* |  |  |  |  | 0.121 |
| 7–12 years old | **2.95 (2.07 – 4.19)** | **2.20 (1.65 – 2.94)** | **1.56 (1.17 – 2.08)** | <0.001 |  |
| 13–15 years old | **2.14 (1.2 – 3.82)** | 1.53 (0.92 – 2.54) | 1.43 (0.87 – 2.38) | 0.001 |  |
| 16–18 years old | 0.75 (0.24 – 2.37) | 0.77 (0.26 – 2.27) | 0.63 (0.21 – 1.85) | 0.241 |  |
| *Resident area* |  |  |  |  | 0.600 |
| Urban | **2.84 (1.96 – 4.14)** | **2.21 (1.62 – 3.02)** | **1.63 (1.2 – 2.23)** | <0.001 |  |
| Rural | **2.03 (1.3 – 3.17)** | **1.57 (1.07 – 2.29)** | 1.21 (0.83 – 1.78) | <0.001 |  |
| *Birthweight* |  |  |  |  | 0.309 |
| ＜4000g | **2.67 (1.94 – 3.66)** | **2.07 (1.59 – 2.7)** | **1.60 (1.23 – 2.08)** | <0.001 |  |
| ≥4000g | 1.55 (0.79 – 3.07) | 1.21 (0.66 – 2.22) | 0.87 (0.47 – 1.59) | <0.001 |  |
| *Breast feeding* |  |  |  |  | 0.155 |
| Yes | **2.40 (1.77 – 3.25)** | **1.94 (1.5 – 2.51)** | **1.52 (1.17 – 1.96)** | <0.001 |  |
| No | **3.00 (1.26 – 7.11)** | 1.65 (0.82 – 3.33) | 1.07 (0.53 – 2.17) | <0.001 |  |
| *Parental highest educational attainment* |  |  |  |  | 0.302 |
| Junior high school or below | **1.92 (1.28 – 2.88)** | **1.69 (1.19 – 2.41)** | 1.33 (0.93 – 1.89) | <0.001 |  |
| Senior high school or above | **3.13 (2.09 – 4.7)** | **2.07 (1.49 – 2.88)** | **1.54 (1.11 – 2.15)** | <0.001 |  |
| *Family history of chronic diseases* |  |  |  |  | 0.508 |
| No | **2.25 (1.46 – 3.47)** | **2.04 (1.45 – 2.89)** | **1.58 (1.12 – 2.23)** | <0.001 |  |
| Yes | **2.46 (1.67 – 3.62)** | **1.76 (1.26 – 2.47)** | 1.33 (0.95 – 1.86) | <0.001 |  |
| *Single children* |  |  |  |  | 0.331 |
| Yes | **2.28 (1.58 – 3.3)** | **1.77 (1.29 – 2.42)** | 1.28 (0.93 – 1.75) | <0.001 |  |
| No | **2.68 (1.7 – 4.22)** | **2.10 (1.43 – 3.06)** | **1.78 (1.22 – 2.6)** | <0.001 |  |
| *Family income* |  |  |  |  | 0.067 |
| < 12000 RMB | **2.57 (1.78 – 3.71)** | **2.03 (1.49 – 2.77)** | **1.43 (1.05 – 1.94)** | <0.001 |  |
| ≥ 12000RMB | **3.86 (1.28 – 11.65)** | 1.80 (0.77 – 4.2) | 2.33 (0.98 – 5.55) | 0.461 |  |
| Don’t know / missing data | **1.99 (1.2 – 3.29)** | **1.73 (1.13 – 2.65)** | 1.32 (0.86 – 2.02) | <0.001 |  |

Notes: The bold values indicated the ORs were statistically significant. The combined unhealthy behaviors of parents were examined as multiple-category independent variable (vs. all behaviors were healthy). Group1, offspring whose parents had 3 unhealthy behaviors; Group2, offspring whose parents had 2 unhealthy behaviors; Group3, offspring whose parents had 1 unhealthy behavior. CVH status of offspring were examined as two categories’ dependent variable (non–ideal vs. ideal CVH status). P for trend values were calculated by including the combined unhealthy behavior of parents as a continuous variable in the model; the interactions between parental unhealthy behaviors and social-demographic factors were examined using the likelihood ratio test, with a comparison of the log likelihood of the two models with and without the interaction terms. Adjusted for provinces, family history of chronic diseases, family monthly income, offspring's sex, age, resident area, birthweight, Single-child status, breast feeding status, and parental highest education attainment.

**Table S8.** Association between single unhealthy behavior of parents and offspring’s different groups of non-ideal CVH status in complete data.

| Parental unhealthy behaviors | 1–3 ideal CVH factors  OR（95% CI） | 4 ideal CVH factors  OR（95% CI） | 5 ideal CVH factors  OR（95% CI） | 6–7 ideal CVH factors  OR（95% CI） |
| --- | --- | --- | --- | --- |
| Parental overweight/obesity  (vs. normal weight) | **1.75 (1.37 – 2.22)** | **1.5 (1.26 – 1.78)** | **1.23 (1.05 – 1.44)** | 1 (reference) |
| Insufficient MVPA  (vs. sufficient) | **1.33 (1.08 – 1.64)** | **1.36 (1.18 – 1.57)** | **1.31 (1.16 – 1.49)** | 1 (reference) |
| Unhealthy dietary behaviors  (vs. healthy) | **2.21 (1.45 – 3.39)** | **1.55 (1.21 – 2.00)** | **1.48 (1.19 – 1.84)** | 1 (reference) |

Notes: The bold values indicated the ORs were statistically significant. A multilevel logistic regression model was used, with pairwise-regression, respectively. Adjusted for provinces, family history of chronic diseases, family monthly income, offspring's sex, age, resident area, birthweight, single-child status, breast feeding status, and parental highest education attainment; CVH, cardiovascular health; MVPA: moderate to vigorous physical activity.

**Table S9**. Association between different levels of single behavior of parents and offspring non-ideal CVH status in complete data.

| Parental lifestyle factors | Non-ideal CVH status | P-trend |
| --- | --- | --- |
| Parental BMI |  | <0.001 |
| < 24 Kg/m^2^ | 1 (Reference) |  |
| 24~27.9 Kg/m^2^ | **1.37 (1.17 - 1.60)** |  |
| ≥28 Kg/m^2^ | 1.22 (0.89 - 1.66) |  |
| Parental MVPA | | <0.001 |
| ≥150 mins/week | 1 (Reference) |  |
| 1–150 min/week | **1.29 (1.11 - 1.51)** |  |
| 0 min/week | **1.38 (1.20 - 1.59)** |  |
| Number of unhealthy dietary factors | | <0.001 |
| 0~1 | 1 (Reference) |  |
| 2~3 | **1.55 (1.27 - 1.89)** |  |
| 4~5 | 1.72 (0.97 - 3.07) |  |

Notes: The bold values indicated the ORs were statistically significant. A multilevel logistic regression model was used. CVH status of offspring were examined as two categories’ dependent variable (non-ideal vs. ideal CVH status). Adjusted for provinces, family history of chronic diseases, family monthly income, offspring's sex, age, resident area, birthweight, single-child status, breast feeding status, and parental highest education attainment. CVH, cardiovascular health; BMI, body mass index; MVPA, moderate to vigorous physical activity.

**Table S10**. Association between combined unhealthy behaviors of parents and offspring’s different groups of non-ideal CVH status in complete data.

| Number of parental unhealthy lifestyle factors | 1–3 ideal CVH factors  OR（95%CI） | 4 ideal CVH factors  OR（95%CI） | 5 ideal CVH factors  OR（95%CI） | 6–7 ideal CVH factors  OR（95%CI） |
| --- | --- | --- | --- | --- |
| 0 | 1 (Reference) | 1 (Reference) | 1 (Reference) | 1 (Reference) |
| 1 | 1.55 (0.84 – 2.87) | 1.4 (0.95 – 2.06) | 1.22 (0.89 – 1.68) | 1 (Reference) |
| 2 | **2.16 (1.17 – 4)** | **1.97 (1.34 – 2.89)** | **1.7 (1.24 – 2.33)** | 1 (Reference) |
| 3 | **4.33 (2.22 – 8.45)** | **3.02 (1.94 – 4.69)** | **1.99 (1.36 – 2.9)** | 1 (Reference) |
| P-Trend | <0.001 | <0.001 | <0.001 | –– |

Notes: The bold values indicated the ORs were statistically significant. A multilevel logistic regression model was used, with pairwise-regression, respectively. P for trend values were calculated by including the combined unhealthy behavior of parents as a continuous variable in the model. Adjusted for provinces, family history of chronic diseases, family monthly income, offspring's sex, age, resident area, birthweight, single–child status, breast feeding status, and parental highest education attainment. CVH, cardiovascular health.

**Table S11.** Association between single parental unhealthy behavior and offspring CVH status with excluding the parents whose BMI<18.5.

| Parental unhealthy behaviors | 1–3 ideal CVH factors  OR（95% CI） | 4 ideal CVH factors  OR（95% CI） | 5 ideal CVH factors  OR（95% CI） | 6–7 ideal CVH factors  OR（95% CI） |
| --- | --- | --- | --- | --- |
| Parental overweight/obesity  (vs. normal weight) | **1.57 (1.28 – 1.92)** | **1.41 (1.22 – 1.64)** | 1.11 (0.98 – 1.27) | 1 (Reference) |
| Insufficient MVPA  (vs. sufficient) | **1.33 (1.11 – 1.59)** | **1.34 (1.18 – 1.52)** | **1.29 (1.16 – 1.44)** | 1 (Reference) |
| Unhealthy dietary behaviors  (vs. healthy) | **1.98 (1.37 – 2.87)** | **1.50 (1.20 – 1.88)** | **1.37 (1.13 – 1.66)** | 1 (Reference) |

Notes: The bold values indicated the ORs were statistically significant. A multilevel logistic regression model was used, with pairwise-regression, respectively. Adjusted for provinces, family history of chronic diseases, family monthly income, offspring's sex, age, resident area, birthweight, single-child status, breast feeding status, and parental highest education attainment; CVH, cardiovascular health; MVPA: moderate to vigorous physical activity.

**Table S12**. Association between different levels of single behavior of parents and offspring non-ideal CVH status with excluding the parents whose BMI<18.5.

| Parental lifestyle factors | Non-ideal CVH status | P-trend |
| --- | --- | --- |
| Parental BMI |  | 0.001 |
| < 24 Kg/m^2^ | 1 (Reference) |  |
| 24~27.9 Kg/m^2^ | **1.26 (1.11 – 1.44)** |  |
| ≥28 Kg/m^2^ | 1.13 (0.88 – 1.45) |  |
| Parental MVPA | | <0.001 |
| ≥150 mins/week | 1 (Reference) |  |
| 1–150 min/week | **1.25 (1.09 – 1.43)** |  |
| 0 min/week | **1.38 (1.22 – 1.56)** |  |
| Number of unhealthy dietary behaviors | | <0.001 |
| 0~1 | 1 (Reference) |  |
| 2~3 | **1.46 (1.22 – 1.74)** |  |
| 4~5 | 1.59 (0.97 – 2.61) |  |

Notes: The bold values indicated the ORs were statistically significant. A multilevel logistic regression model was used. CVH status of offspring were examined as two categories’ dependent variable (non-ideal vs. ideal CVH status). Adjusted for provinces, family history of chronic diseases, family monthly income, offspring's sex, age, resident area, birthweight, single-child status, breast feeding status, and parental highest education attainment. CVH, cardiovascular health; BMI, body mass index; MVPA, moderate to vigorous physical activity

**Table S13**. Association between combined unhealthy behaviors of parents and offspring’s different groups of non-ideal CVH status with excluding the parents whose BMI<18.5.

| Number of parental unhealthy lifestyle factors | 1–3 ideal CVH factors  OR（95%CI） | 4 ideal CVH factors  OR（95%CI） | 5 ideal CVH factors  OR（95%CI） | 6–7 ideal CVH factors  OR（95%CI） |
| --- | --- | --- | --- | --- |
| 0 | 1(Reference) | 1(Reference) | 1(Reference) | 1(Reference) |
| 1 | 1.68 (0.95 – 2.95) | **1.48 (1.05 – 2.09)** | 1.16 (0.88 – 1.53) | 1(Reference) |
| 2 | **2.26 (1.29 – 3.97)** | **2.02 (1.43 – 2.85)** | **1.52 (1.16 – 2.00)** | 1(Reference) |
| 3 | **3.93 (2.15 – 7.17)** | **2.85 (1.94 – 4.18)** | **1.7 (1.24 – 2.35)** | 1(Reference) |
| P-Trend | <0.001 | <0.001 | <0.001 | –– |

Notes: The bold values indicated the ORs were statistically significant. A multilevel logistic regression model was used, with pairwise-regression, respectively. P for trend values were calculated by including the combined unhealthy behavior of parents as a continuous variable in the model. Adjusted for provinces, family history of chronic diseases, family monthly income, offspring's sex, age, resident area, birthweight, single-child status, breast feeding status, and parental highest education attainment. CVH, cardiovascular health.
